# Participation of the *Salmonella* OmpD Porin in the Infection of RAW264.7 Macrophages and BALB/c Mice

**DOI:** 10.1371/journal.pone.0111062

**Published:** 2014-10-31

**Authors:** Francisco Ipinza, Bernardo Collao, Debbie Monsalva, Victor H. Bustamante, Roberto Luraschi, Melissa Alegría-Arcos, Daniel E. Almonacid, Daniel Aguayo, Iván L. Calderón, Fernando Gil, Carlos A. Santiviago, Eduardo H. Morales, Edmundo Calva, Claudia P. Saavedra

**Affiliations:** 1 Laboratorio de Microbiología Molecular, Departamento de Ciencias Biológicas, Facultad de Ciencias Biológicas, Universidad Andres Bello, Santiago, Chile; 2 Departamento de Microbiología Molecular, Instituto de Biotecnología, Universidad Nacional Autónoma de México, Cuernavaca, México; 3 Center for Bioinformatics and Integrative Biology, Facultad de Ciencias Biologicas, Universidad Andres Bello, Santiago, Chile; 4 Laboratorio de Microbiología, Departamento de Bioquímica y Biología Molecular, Facultad de Ciencias Químicas y Farmacéuticas, Universidad de Chile, Santiago, Chile; 5 Great Lakes Bioenergy Research Center and Department of Biomolecular Chemistry, University of Wisconsin-Madison, Madison, Wisconsin, United States of America; New York State Dept. Health, United States of America

## Abstract

*Salmonella* Typhimurium is the etiological agent of gastroenteritis in humans and enteric fever in mice. Inside these hosts, *Salmonella* must overcome hostile conditions to develop a successful infection, a process in which the levels of porins may be critical. Herein, the role of the *Salmonella* Typhimurium porin OmpD in the infection process was assessed for adherence, invasion and proliferation in RAW264.7 mouse macrophages and in BALB/c mice. In cultured macrophages, a Δ*ompD* strain exhibited increased invasion and proliferation phenotypes as compared to its parental strain. In contrast, overexpression of *ompD* caused a reduction in bacterial proliferation but did not affect adherence or invasion. In the murine model, the Δ*ompD* strain showed increased ability to survive and replicate in target organs of infection. The *ompD* transcript levels showed a down-regulation when *Salmonella* resided within cultured macrophages and when it colonized target organs in infected mice. Additionally, cultured macrophages infected with the Δ*ompD* strain produced lower levels of reactive oxygen species, suggesting that down-regulation of *ompD* could favor replication of *Salmonella* inside macrophages and the subsequent systemic dissemination, by limiting the reactive oxygen species response of the host.

## Introduction

Porins are outer membrane proteins organized as homotrimers, homodimers and monomers that form aqueous channels that allow passive transport of hydrophilic molecules of low molecular weight (<600 Da) [1], [2]. These properties of porins confer a dual role that enable bacteria to interact with the extracellular environment by allowing the entrance of nutrients and also the excretion of waste products and toxic substances [3], [4]. Among the major porins in *Salmonella enterica* serovar Typhimurium (*S*. Typhimurium) are OmpF, OmpC, PhoE and OmpD. OmpF and OmpC are mainly involved in the transport of cations, whereas PhoE is involved in the transport of anions [5]. During the last years, evidence has emerged from our laboratory that illustrate some roles for the porins. We previously demonstrated that OmpD and the minor porin OmpW facilitate the uptake of hydrogen peroxide (H_2_O_2_) and/or hypochlorous acid (HOCl) [6], [7], two oxidizing molecules produced in the phagocytic oxidative burst. Porins may also act as virulence factors. For instance, OmpCD and M35 from *Moraxella catarrhalis* are involved in adhesion to host cells [8], [9]; OmpK36 from *Klebsiella pneumoniae* and OmpC from *Shigella flexneri* are involved in the invasion stage of infection [10], [11] and OmpC from *Escherichia coli* triggers a serum bactericidal response through an antibody-dependent pathway [12]. In *S*. Typhimurium, OmpC and OmpF porins are recognized by macrophages, participate in phagocytosis and trigger a signaling cascade which regulates the ROS response by the host and the phagolysosomal maturation [13], [14]. Furthermore, mutants in the *S*. Typhimurium *ompS1* and *ompS2* genes are attenuated for virulence in a mouse model [15].

OmpD is the most abundant porin in the outer membrane of *S*. Typhimurium: its abundance increases in response to anaerobiosis and decreases at low pH and under oxidative stress, conditions that are present inside the host during infection. Furthermore, it is involved in the efflux of toxic compounds and, interestingly, the *ompD* gene is not present in the *S*. Typhi genome, which could be related to the particular characteristics of the *S*. Typhimurium infective cycle [4], [7], [16], [17]. Regarding the role of OmpD in virulence, Dorman *et al*. (1989) [18] found that a mutation in the *ompD* gene slightly reduced the virulence of *S*. Typhimurium in BALB/c mice as revealed by LD_50_ assays. However, Meyer *et al*. (1998) [19] did not find significant differences in virulence between an *ompD* mutant and its corresponding wild-type strain, neither in the murine model nor in tissue culture. Other studies have shown that OmpD is involved in adherence and recognition of *S*. Typhimurium to human macrophages and intestinal epithelial cells, during the initial stages of infection [13], [20]. Moreover, studies based on the B1b cell antibody response showed that OmpD is a key factor in the humoral response in mice [21].

Since the role of OmpD as a virulence factor is still controversial, in this work we evaluated its effect in the adherence, invasion and proliferation of *S*. Typhimurium in RAW264.7 macrophages. The results obtained with these assays were then compared with those addressed in the murine model. A Δ*ompD* strain exhibited increased invasion and proliferation in macrophages, in agreement with the increased ability of this mutant for survival and replication in the mouse model. Interestingly, the *ompD* transcript levels diminished in the wild-type strain infecting macrophages and BALB/c mice. Additionally, the levels of ROS generated by macrophages infected with *S.* Typhimurium were directly related to the presence of the OmpD porin. In conclusion, the results presented here indicate that *S*. Typhimurium has the ability to down-regulate the expression of *ompD* in order to improve its survival inside the host, thus allowing dissemination and the establishment of a systemic infection. Some aspects concerning these observations are discussed below.

## Materials and Methods

Ethics Statement. This study was carried out in strict accordance with the recommendations in the Guide for the Care and Use of Laboratory Animals of the National Institutes of Health. Futhermore, all animal work and care was carried out in strict accordance with the guidelines approved by the Bioethic Committee of Universidad Andres Bello, Santiago, Chile (Vicerrectoría de Investigación y Postgrado, Universidad Andres Bello) for FONDECYT grant #1085131.

### 2.1. Bacterial strains and culture conditions

Bacterial strains used in the present study are described in Table 1. Bacterial cultures were grown in Luria-Bertani (LB) broth at 37 °C with shaking at 200 rpm. When required, LB was supplemented with ampicillin (Amp, 100 µg/ml) or kanamycin (Kan, 50 µg/ml). To induce overexpression of OmpD or OmpW from the corresponding recombinant plasmids, the culture medium was supplemented with 10 mM arabinose.

**Table 1:** Bacterial strains used in this study.

Strains	Relevant characteristic(s)	Source or reference
***S*** **. Typhimurium**		
14028s	Wild-type strain (ATCC)	G. Mora
Δ*ompD*	Δ*ompD*::*kan*	[3]
Δ*ompD*/pBAD-*ompD*	Δ*ompD*::*kan* transformed with a derivative of pBAD vector carrying the *S*. Typhimurium *ompD* gene	This work
Δ*ompD*/pBAD	Δ*ompD*::*kan* transformed with an empty pBAD vector	This work
Δ*ompW*	Δ*ompW*::*kan*	[3]
Δ*ompW*/pBAD-*ompW*	Δ*ompW*::*kan* transformed with a derivative of pBAD vector carrying the *S*. Typhimurium *ompW* gene	[3]
Δ*ompW*/pBAD	Δ*ompW*::*kan* transformed with an empty pBAD vector	This work
Δ*ssrB*	Δ*ssrB*::*kan*	E. Calva
***E. coli***		
TOP10	F^-^ *mcrA* Δ(*mrr*-*hsdRMS*-*mcrBC*) φ80*lacZ*ΔM15 Δl*acX74 nupG recA1 araD139* Δ(*ara-leu*)7697 *galE*15 *galK*16 *rpsL*(Str^R^) *endA1* λ^-^	Invitrogen
TOP10/pBAD-*ompD*	TOP10 transformed with a derivative of pBAD vector carrying the *S.* Typhimurium *ompD* gene	This work
TOP10/pBAD-*ompW*	TOP10 transformed with a derivative of pBAD vector carrying the *S.* Typhimurium *ompW* gene	This work

### 2.2 Infection models

#### 2.2.1. Macrophage infection assays

An *in vitro* gentamicin protection assay based on the method described by Lissner *et al*. [22] was used to measure invasion and intracellular proliferation of *S.* Typhimurium wild-type strain 14028 s and its Δ*ompD* and Δ*ompW* derivative mutants in RAW 264.7 murine macrophages (ATCC Number: TIB-71). RAW 264.7 macrophages were grown at 37 °C with 5% CO_2_ in RPMI supplemented with 10% fetal bovine serum (FBS) (Hyclone). On the other hand, bacteria were grown under microaerophilic conditions in LB medium at 37 °C to an OD_600_ of ∼0.2. Monolayers of RAW 264.7 macrophages in 96-wells plates were infected with approximately 5×10^4^ colony-forming units (CFU) in 0.1 mL of phosphate-buffered saline (PBS) at a multiplicity of infection (MOI) of 100 bacteria per cell, and co-incubated for 1 h at 37 °C with 5% CO_2_. The monolayers were washed twice with PBS, and then with 100 µL of RPMI +10% FBS containing 50 µg/ml gentamicin. Samples from three wells were lysed with sodium deoxycholate (0.5% in PBS). CFU were determined from infected macrophages at different time points, representing the different stages of infection: adherence (1 h), invasion (2 h) and proliferation (4, 6, 8, 10 and 12 h), and the percenteges were calculated as below.

100× CFU at t_y_/CFU at t_x_ for the mutant strain/CFU at t_y_/CFU at t_x_ for the wild type strain. For adherence, invasion and proliferation CFU at t_x_ were obtained from the bacterial inoculum used to infect macrophages, from bacteria adhered to macrophages and from bacteria that invaded to macrophages, respectively; whereas CFU at t_y_ were obtained at the indicated times post infection used to evaluate each of these phenotypes.

#### 2.2.2. Determination of ROS levels inside macrophages

To determine the intracellular levels of ROS in murine macrophage cells, the oxidant-sensitive probe Diclorodihydrofluorescein diacetate (H2DCFDA) [24] was used. Cells were grown and infected as described above for the macrophage infection assays. The cells from the culture infection were lysed by adding sodium deoxycholate (0.5% in PBS). After lysis, 5 µl of 10 µM H2DCFDA were added for detecting intracellular total ROS levels. Fluorescence intensity was measured every h for 12 h using a Turner BioSystems TBS-380 fluorimeter (excitation, 490 nm; emission, 519 nm) and the measurement was normalized by µg of protein.

#### 2.2.3. Mice infection assays

Animals. BALB/C female mice were used (7 to 8 week-old). All animals were allocated to metallic cages and kept in a temperature controlled environment (22–24 °C) in animal facilities for infected mice.

*Competitive index (CI)*. CI assays were performed as described by Segura *et al*. [25] and Rodríguez-Morales *et al*. [15]. Briefly, bacteria were grown in LB with shaking to an OD_600_ of 0.6. The wild-type 14028 s strain and each of its mutant derivatives were mixed in a 1∶1 ratio up to 10^3^ bacteria in 0.1 ml of PBS. Oral and intraperitoneal infections were performed using groups of four to eight female BALB/c mice (7 to 8 week-old). Animals were monitored twice a day (morning and evening) for signs of intoxication or death. Briefly, the animals were monitored for changes in weight, general aspect, spontaneous behavior, and response to manipulation. In order to avoid unnecessary pain, animals with signs of suffering were immediately euthanized by cervical dislocation. Liver and spleen were harvested at days 3 and 5 post infection. The organs were homogenized in 5 ml of DPBS (Dulbecco's Phosphate-Buffered Saline) and then lysed by the addition of sodium deoxycholate (0.5% in PBS) and incubation for 15 min at room temperature. CFU were determined by plating serial 10-fold dilutions of cell lysates onto LB agar plates with or without 50 µg/ml Kan. CIs were calculated as previously described [25].

*Bacteremia*. To determine bacteremia, groups of six female BALB/c mice (7 to 8 week-old) were orally infected with 10^3^ CFU of wild-type or mutant strains in 0.1 ml PBS, which represent the same doses used in CI assays. The animals health condition was monitored twice a day (morning and evening) for signs of intoxication or death as described for the competitive index experiments. To avoid unnecessary pain, animals with signs of suffering were euthanized as previously described. After 3 and 5 days of infection, blood samples (100 µl) were obtained aseptically from the tail vein. CFU were determined by plating serial 10-fold dilutions of blood samples onto LB agar plates without or with 50 µg/ml Kan, which were then incubated at 37 °C overnight [26].

### 2.3. Construction of plasmids

The *S*. Typhimurium *ompD* and *ompW* genes were amplified by PCR, cloned into pBAD-TOPO TA [23] and transformed into *E. coli* TOP10. Primer sequences were 5′ATGAAACTTAAGTTAGTGGC3′ (pBADompDFw) and 5′GAACTGGTAGTTCAGACCAA3′ (pBADompDRv); 5′ATGAAAAAATTTACAGfTGGC3′ (pBAD-ompWFw); 5′GAAACGATAGCCTGCCGAGA3′ (pBAD-ompWRv). Plasmids pBAD-*ompD* or pBAD-*ompW* were extracted from *E. coli* TOP10 and transfered to *S*. Typhimurium strains through electroporation of competent cells.

### 2.4. Detection of *ompD* transcript levels by quantitative real-time RT-PCR (qRT-PCR)

RAW264.7 macrophages were infected with strains Δ*ompD*, Δ*ompW* or 14028 s before being lysed with sodium deoxycholate (0.5% in PBS) to obtain the samples for RNA extraction. Also, liver and spleen from infected BALB/c mice were extracted and processed with a T-10 Basic Ultra-Turrax (IKA) homogenizer and samples were stored at 4 °C for subsequent RNA extraction. Total RNA was extracted using the RNeasy kit (Qiagen) following the manufacturer's instructions. The RNA was treated with 2 U of DNase I (Roche) for 1 h to remove trace amounts of DNA. cDNA synthesis was carried out at 37 °C for 1 h in 25 µl of a mixture containing 2.5 pmol of the specific reverse primers, 10 µl of template RNA (5 µg), 0.2 mM of dNTPs, 1 µl of sterile water, 4 µl of 5× buffer (250 mM Tris-HCl pH 8.3, 375 mM KCl, 15 mM MgCl_2_, 10 mM DTT), 40 U of RNasin and 200 U of MMLV reverse transcriptase (Invitrogen).

The PCR reaction mix contained 10 µl of template cDNA, 5 µl of 10× buffer (200 mM Tris-HCl pH 8.4, 500 mM KCl), 2 mM MgCl_2_, 10 mM dNTPs, 10 pM of each primer, and 3 U of Taq polymerase (Invitrogen) in a final volume of 50 µl. PCR was performed following initial denaturation at 95 °C for 5 min and then 25 cycles of 95 °C for 60 s, 50 °C for 60 s and 72 °C for 60 s followed by a final 10 min extension step at 72 °C. RT-PCR of 16S rRNA was carried out under the same conditions as the control. Amplification products were fractionated by electrophoresis using 1% agarose gels. The specific primers used were 5′ TGTTGCCACCTACCGTAACA 3′ (*ompD*Fw) and 5′ GGTCGCCAGGTAGATGTTGT 3′ (*ompD*Rv) for the *ompD* gene; 5′ ATGAAAAAATTTACAGTGG 3′ (*ompW*_RT_Fw) and 5′ GAAACGATAGCCTGCCGA 3′ (*ompW*_RT_Rv), for the *ompW* gene 5′ GTAGAATTCCAGGTGTAGCG 3′ (16SFw) and 5′ TTATCACTGGCAGTCTCCTT 3′ (16SRv) for the 16S rRNA gene (16S). Relative quantification of *ompD*, *ompW* and 16S transcript levels by qRT-PCR was performed using the Brilliant II SYBR Green QPCR Master Reagent kit and the Mx3000P detection system (Stratagene). 16S rRNA levels were used for normalization. The qRT-PCR mixture (20 µl) contained 1 µl of cDNA template and 120 nM of each primer (*ompD*Fw and *ompD*Rv). The qRT-PCR was performed under the following conditions: 10 min at 95 °C followed by 40 cycles of 30 s at 95 °C, 45 s at 58 °C and 30 s at 72 °C. A previous standard quantification curve with serial dilutions of RT-PCR products was constructed for each gene to calculate the amplification efficiency. These values were used to obtain the ratio between the gene of interest and the expression of the 16S rRNA gene as described by Pfaffl [27].

### 2.5. Sequence similarity network (SSN) of the Omp superfamily

To generate the SSN, 110 Omps annotated in Uniprot [28] were used as seed sequences (Q05811, Q83EK8, B2SAB9, Q2YMY8, A9MA14, Q7CNU3, Q45325, Q45331, Q44620, P0DI94, Q45079, P0DI93, Q44665, Q2YMY7, Q44619, A9MA15, Q8YG56, Q45078, Q45324, Q45330, A5VPH9, B0CKW5, P0DI96, P0DI95, Q9PLL3, Q6RYW5, A3M8K2, P24305, Q9Z752, P0A0V2, P07050, P0A0V3, P38006, P43840, P38368, P45996, P13415, P18194, Q05146, P24016, P0A911, P0A910, B7LNW7, P0C8Z2, P24754, P24017, P09146, P05430, P57041, E6MXW0, P0DH58, Q8Z7S0, P02936, P04845, P24755, P0DJO6, P02935, I2BAK7, Q8ZG77, P38399, P30690, P30687, P18195, P30688, P30689, P30691, E6MZM0, P20148, P57042, P30692, Q8XE41, Q8CVW1, P06996, Q48473, P09888, O33507, P0A264, Q54471, P0A263, A0RZH5, P02931, Q56113, O33980, Q56828, P37432, P76045, O54339, O54340, P29739, Q9CNN9, P77747, Q56110, Q56111, A5F934, Q8DBX0, Q87LZ1, P0C6Q6, P20149, P46026, P46027, Q48216, P43839, Q48218, Q48221, P46025, Q48217, Q48219, Q48220, P80672, P38369). To avoid redundance in the searches, the 110 seed sequences were clustered using CD-HIT [29] at 50% sequence identity, resulting in 25 clusters. A representative member of each cluster was used to perform a search using blastp [30] against a local nr database downloaded from the NCBI ftp site on May 2014. Only proteins with an E-value lower than 1e^−5^ for at least two of the representative sequences in the blastp search were further considered (24,949 sequences). In order to visualize the results, the full set of sequences were clustered at 70% sequence identity using CD-HIT, resulting in 6,223 clusters. For simplicity, only the clade including OmpD was further analyzed, consisting of 1,141 clusters, representing in total 8275 proteins. The largest sequence in each cluster was used as a representative to generate a sequence similarity network. Since blastp E-values are not symmetrical, the searches were performed using one of the sequences under comparison as query and the other as subject and *vice versa*, and the worst reciprocal blastp E-value for each pair was selected. The results were visualized using the organic layout in Cytoscape 2.8.3 [31] at different thresholds for the E-value.

### 2.6. OmpD Homology Modelling and Surface Electrostatic Potential Comparisons

The 3D model of OmpD was constructed using Modeller 9.12 [32]. The structures of OmpF from *Salmonella Typhi* (PDB: 3NSG) and *E. coli* (PDB: 2ZFG), and OmpC from *E. coli* (PDB: 2J1N) and *Salmonella Typhi* (PDB: 3UU2) were used as three-dimensional templates. The STAMP algorithm was used to obtain a reference multiple alignment by 3D-structure comparison [33]. Several OmpD trimers models were generated and evaluated by their DOPE potentials, from which the model with the best score was selected and presented herein. All porins were three-dimensionally aligned using the STAMP algorithm and their electrostatic surface potentials were calculated using APBS [34] and visualized in VMD [35] using the MSMS solvent excluded surface calculator [36].

### 2.7. Statistical analysis

Statistical analyses were performed using Prism software (Graph Pad) and p values of ≤0.05 were considered significant in a minimum of three independent experiments biological and technical replicates. The statistical analyses used the one- or two-way ANOVA with a post-hoc Bonferroni test, or t-test. The type of analysis used in each experiment is indicated in the corresponding Figure legend.

## Results

### 3.1. OmpD affects *Salmonella* lifestyle inside macrophages

**Figure 1:**
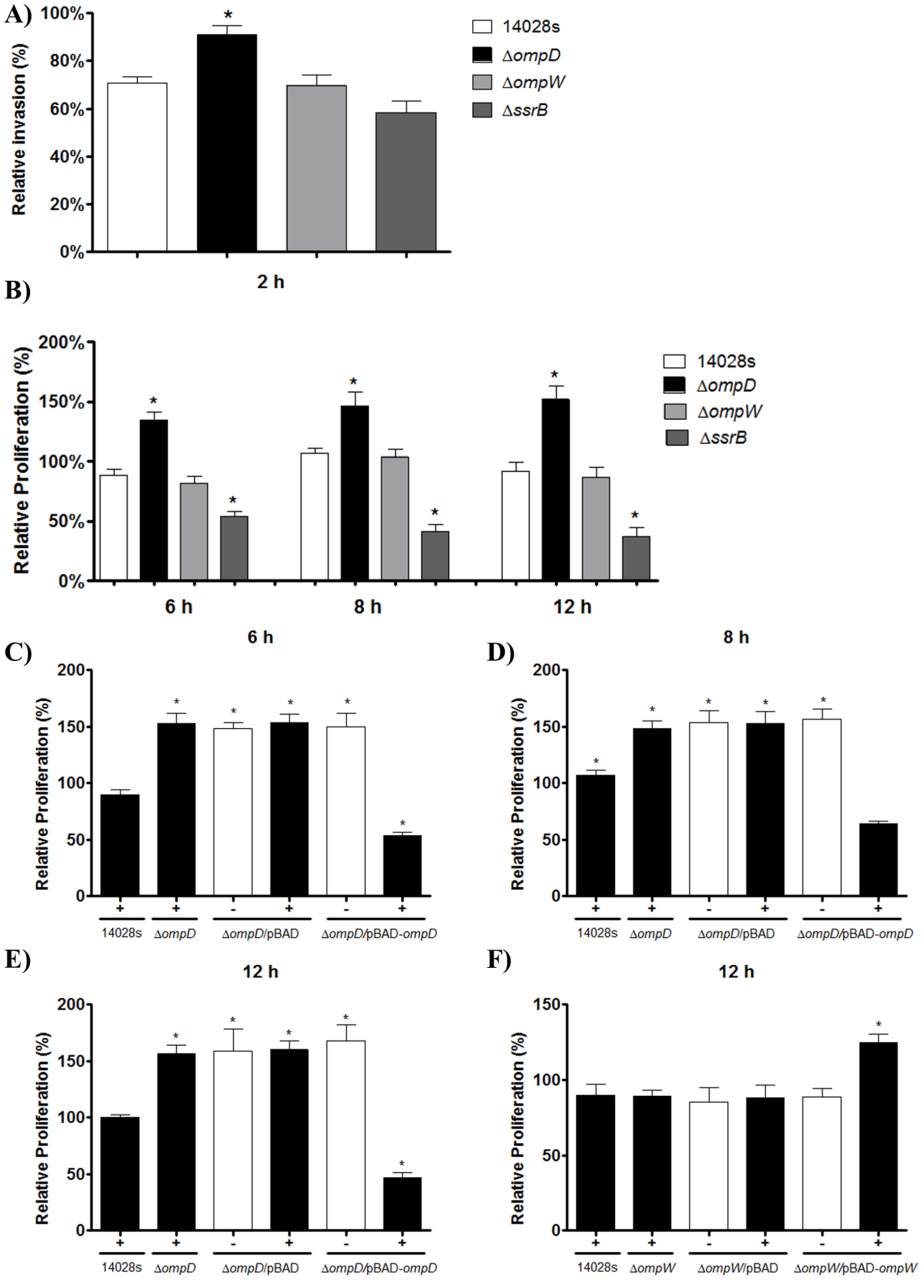
Effect of OmpD in the proliferation of *S*. Typhimurium inside macrophages. Murine RAW 264.7 macrophages were infected (MOI of 100∶1) with *S*. Typhimurium 14028 s, and its Δ*ompD*, Δ*ompW* and Δ*ssrB* derivative mutants, containing or not the pBAD vector, or the plasmids pBAD-*ompD* or pBAD-*ompW* that overexpress OmpD and OmpW, respectively. CFU of the different strains were determined after recovery from infected macrophages at the indicated time points. The relative percentage of invasion at 2 h post infection (A) and of proliferation at 6, 8 and 12 h post infection (B) was determined to analyze the effect of the absence of *ompD*, *ompW* or *ssrB* genes. The relative percentage of proliferation at 6 (C), 8 (D) and 12 h (E) post infection was determined to analyze the effect of the absence and overexpression of OmpD. The relative percentage of proliferation at 12 h (F) post infection was determined to analyze the effect of the absence and overexpression of OmpW. Asterisks represent significant statistical difference between 14028 s and mutants used in this study (* *p*≤0.05). Values are mean ± SD.

In order to evaluate the participation of OmpD in the adhesion, invasion and proliferation of *S*. Typhimurium in host cells, infection assays with macrophages were performed using the 14028 s wild-type and its isogenic Δ*ompD* strain. Since SsrB is a transcriptional regulator required for the expression of the SPI-2 genes, which are essential for the intracellular proliferation of *Salmonella* and thus for its systemic infection in mice [37], a Δ*ssrB* strain was used in these assays as a negative control. On the other hand, to compare the effect of OmpD with that of another porin, the Δ*ompW* strain was also tested. No significant differences in the adherence were observed between all the strains tested (Figure S1); however, the Δ*ompD* strain showed an increase of about 20% and 35% in invasion and proliferation, respectively, in comparison to the wild-type strain (Figure 1A & B). In contrast, the Δ*ompW* strain showed invasion and proliferation patterns comparable to the wild-type strain and, as expected, the Δ*ssrB* strain showed a decreased proliferation phenotype (Figure 1A & B).

Since the absence of OmpD increased the ability of *S*. Typhimurium to invade and proliferate inside host cells, we hypothesized that overexpression of OmpD could have the opposite effect. Hence, macrophage infection assays with the Δ*ompD* strain harboring the pBAD-*ompD* vector were performed. The overexpression of OmpD from pBAD-*ompD* drastically decreased (around 85%) the hyper-proliferation of the Δ*ompD* strain at 6, 8 and 12 h post-infection, to levels even lower than those shown by the wild-type strain (Figure 1C-E). In contrast, the overexpression of OmpW from pBAD-*ompW* had a positive effect in the intracellular proliferation of the Δ*ompW* strain (Figure 1F). Furthermore, OmpD or OmpW overexpression did not significantly affect the adherence and invasion phenotypes (data not shown). Together, these results indicate that the presence of OmpD has a detrimental effect on the ability of *S*. Typhimurium to survive and replicate inside macrophages, suggesting that the bacterium might be negatively regulating the expression of *ompD* inside macrophages to increase its chances of success in terms of infection.

### 3.2. *ompD* is down-regulated in *Salmonella* residing in macrophages

**Figure 2:**
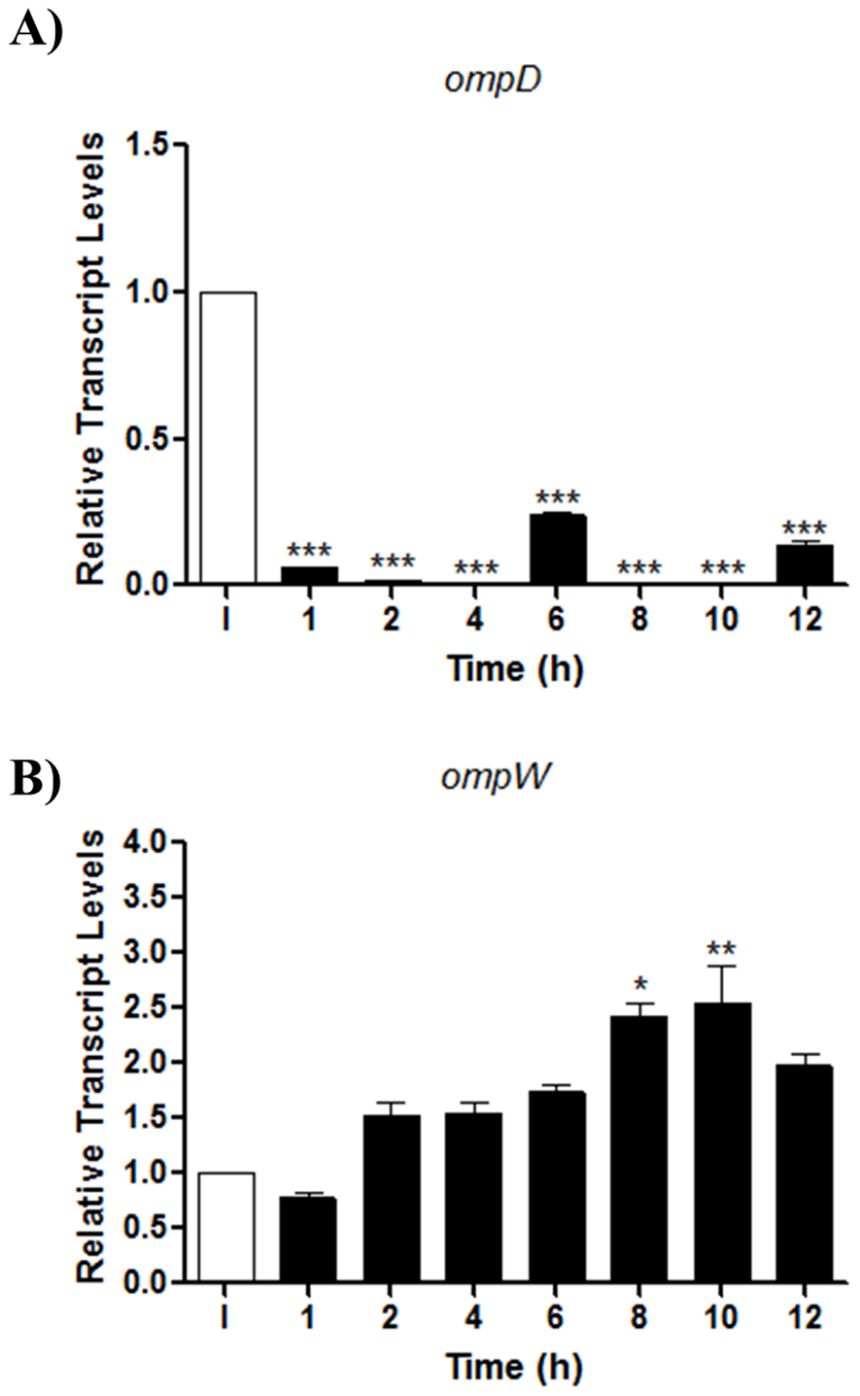
Expression of *S.* Typhimurium 14028 s *ompD* inside macrophages. Transcript levels of *ompD* (A) and *ompW* (B) from strain 14028 s were quantified by qRT-PCR by recovering total RNA from the infected macrophages at 1 to 12 h post infection, and from bacteria of the inoculating culture (I). Experiments were performed in triplicate. Asterisks represent significant statistical differences between the control and mutants strains (* *p*≤0.05; ** *p*≤0.005;*** *p*≤0.001). Values are mean ± SD.

In order to determine whether *ompD* expression is repressed when *S*. Typhimurium resides inside macrophages, the *ompD* mRNA levels were determined by qRT-PCR in macrophages infected with the wild-type strain at 1 to 12 h post-infection. Transcript levels of *ompD* obtained from infected macrophages were compared to those from the bacterial inoculum used for infecting cells. As shown in Figure 2A, the *ompD* mRNA levels were significantly decreased at all times assayed. In contrast, the *ompW* transcript levels were slightly induced at early times post-infection, which was more evident at 8 and 10 h post-infection (Figure 2B). In agreement with these results, a previous transcriptomic study also showed down-regulation and induction of the *ompD* (*nmpC*) and *ompW* transcript levels, respectively, when *Salmonella* is inside macrophages [38]. Thus, repression of *ompD* could be necessary for efficient intracellular proliferation of *Salmonella*.

### 3.3. ROS levels are diminished in macrophages infected with the *Salmonella *Δ*ompD* strain

**Figure 3:**
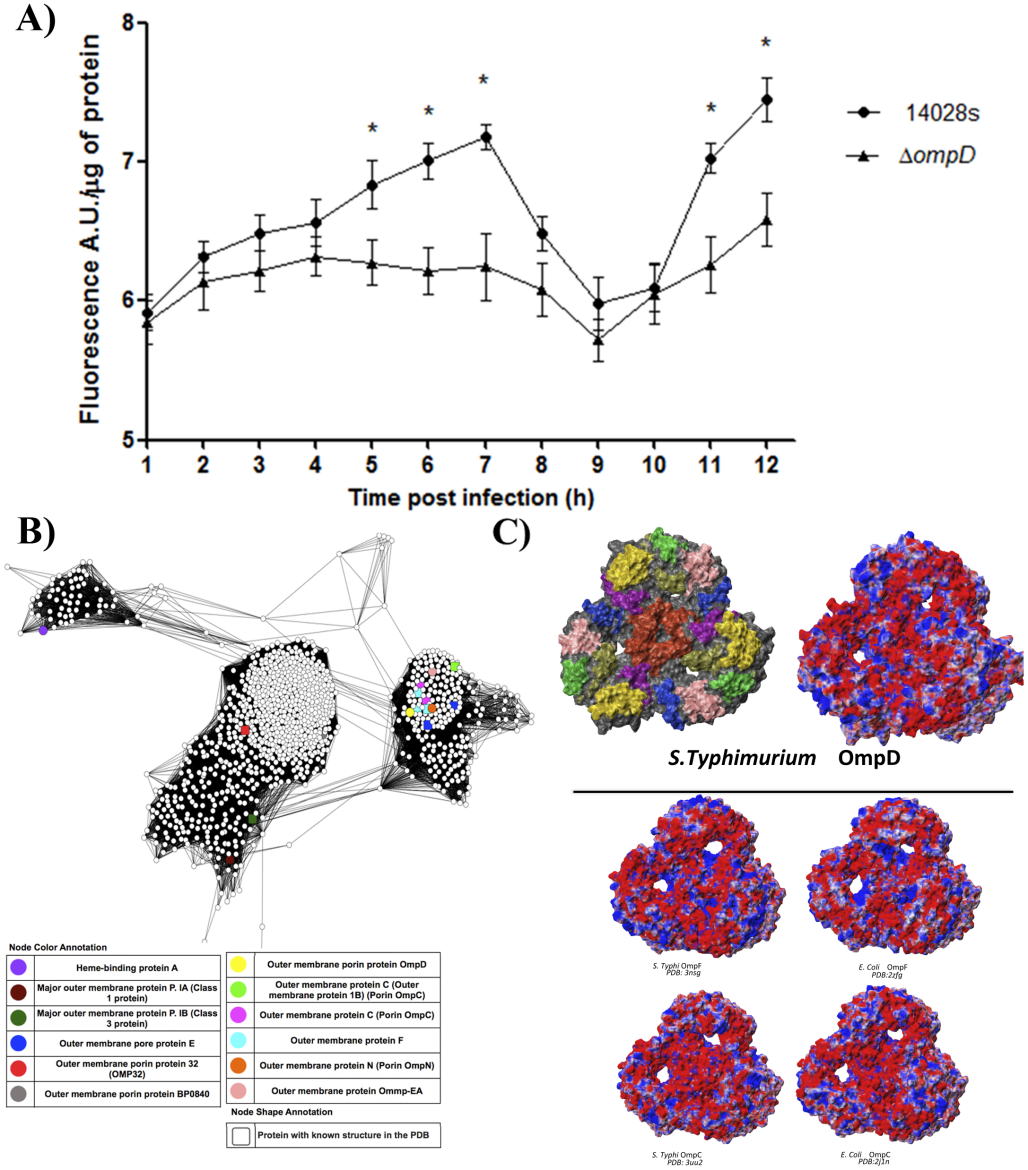
Intracellular levels of total ROS in RAW 264.7 macrophages infected and similarity between OmpD and other members of the OMP superfamily. (A) Total ROS were determined at 1 to 12 h post infection in macrophages infected with strains 14028 s and Δ*ompD* using the oxidant-sensitive probe H2DCFDA (fluorescence adjusted per µg of total protein of the sample). Asterisks represent statistical significant differences (* p≤0.05). A.U.  =  arbitrary units. (B) Sequence similarity network of OmpD and its closest homologues in the Omp superfamily. Nodes represent protein sequences, and edges represent worst reciprocal blastp E-values that are higher than a given threshold. Visualization was performed using the organic layout in Cytoscape 2.8.3 [Cline et al. 2007]. Reviewed proteins from Uniprot that have evidence of existing at the protein level are shown in color. Squares correspond to proteins that have known crystal structures in the Protein Data Bank. Edges filtered to e-value <1e-14, median alignment length: 341 residues, median identity: 34.0%. (C) Electrostatic surface potential of the biological assemblies of OmpD and SLAM-recognized proteins OmpC and OmpF. c.1) Periplasmic loops are mapped into the molecular surface: L1 blue, L2 red, L3 violet, L4 orange, L5 yellow, L6 ochre, L7 green and L8 Pink; c.2) Calculated surface electrostatic potential of OmpF from *Salmonella* Typhi (PDB: 3nsg); OmpF from *E. coli* (PDB:2zfg); OmpC from *Salmonella* Typhi (PDB: 3uu2) and OmpC from *E. coli* (PDB:2j1n) contoured at +- 2.0 kT.

Previously, we reported that *ompD* expression is down-regulated by exposure of *S*. Typhimurium to H_2_O_2_ [7]. As this down-regulation of *ompD* expression is also observed inside macrophages, we investigated whether the presence of this outer membrane protein influences the levels of ROS produced by macrophages during infection. Total ROS levels were determined in macrophages infected with the wild-type and Δ*ompD* strains at 1 to 12 h post-infection. As shown in Figure 3A, the macrophages infected with the Δ*ompD* strain evidenced significantly lower levels of total ROS than the macrophages infected with the wild-type strain. This difference was not observed between macrophages infected with Δ*ompW* and the wild-type strain (data not shown). Despite this observation, a drastic decrease of total ROS at 7 to 9 h post-infection occurred (Figure 3A), probably explained by the sequential roles of oxidative and nitrosative species produced coordinately in a temporal relationship by an early NADPH oxidase and the late inducible nitric oxide synthase (iNOS), respectively [39], [40].

### 3.4. *In silico* comparisons of OmpD and other members of the Omp superfamily

To shed light on the mechanism by which OmpD impacts ROS production by macrophages, and since *E. coli* OmpC and OmpF play a similar role, an *in silico* structural analysis was performed by comparing OmpD with the aforementioned porins. To rationalize the findings obtained for OmpD as a member of the Omp superfamily, a sequence similarity network (SSN) [41] was generated. SSNs are networks where nodes correspond to the sequence of proteins and edges to blastp E-values between the sequences connected by the edge, representing a fast way to compute and visually summarize groups of proteins based on all-against-all sequence comparisons. SSNs are very robust and allow to easily identify clades and they also correlate well with phylogenetic trees [41], [42], [43]. Figure 3B shows a SSN of OmpD and its closest homologues from NCBI's nr database, theresholded at an E-value of 1e^−14^, which corresponds to the highest E-value where all members of the superfamily in the network are connected to at least one other node. Figure S2 shows the same network thersholded at an E-value of 1e^−155^, corresponding to the highest E-value where OmpD is still connected to other known superfamily members. In total, 8,275 proteins were clustered at 70% identity resulting in 1,141 clusters, with the largest porin in each cluster used as nodes to generate the SSN. When only edges that have E-values smaller than 1e^−14^ were considered (Figure 3B, median alignment length of 341 residues, median identity 34.0%), OmpD forms a clade together with the OmpC, OmpE, OmpF and OmpN porins. At a more stringent E-value threshold of 1e^−155^ (Figure S2, median alignment length of 380 residues, median identity 65.2%), OmpD is about to form an independent clade, with its closest homologues being OmpC and OmpN, but still close to OmpE and OmpF. Given that OmpC and OmpF are recognized through a member of the SLAM receptor family, we hypothesize that OmpD may be involved in a similar recognition mechanism, which would explain the fact that macrophages infected with the Δ*ompD* strain evidenced significantly lower levels of total ROS.

To further investigate this recognition mechanism by SLAM, we performed multiple sequence alignments of OmpD with OmpC and OmpF from *E. coli* and *S.* Typhi. Interestingly, conserved amino acids are located in key positions at the loop regions (data not shown), in agreement with the loop-sequence alignment proposed by Berger et al. One of the main characteristics of SLAM is that it has a two-layered β-sheet structure, where the front sheets provide a positive surface electrostatic potential that acts as a complementary interface for receptor binding. Based on this information, we hypothesized that OmpD has similar surface properties as OmpF and OmpC, which are recognized by SLAM receptors [14]. To evaluate the surface properties of these porins, which could participate on SLAM-binding, we determined the electrostatic surface potential of OmpC and OmpF crystallographic structures and, as its atomic structure is not resolved, an OmpD 3D homology-based model. OmpD, OmpC and OmpF monomers have distinctive surface electrostatic potentials with a predominant conservation on surface areas corresponding to the sequences of Loop 2 (L2) and Loop 5 (L5). Interestingly, the L2 surface is predominantly positive in the monomeric state, which could impede the binding to SLAM due to an electrostatic impediment. However, when porins are organized as trimers (biological state), Loop 2 connects neighbour subunits generating a negative solvent-exposed surface at the center of the trimer (Figure 3C). The surface potential of members of the OmpF family has a positive potential located at the surface near the pore lumen, while in members of the OmpC family the positive charges are dispersed throghout the surface. Similarly to what was found in the SSN (Figure 3B), the surface of OmpD has properties more similar to the OmpC family, as it has a negative electrostatic potential with several positive charges delocalized at the trimer surface.

### 3.5. The *Salmonella* Δ*ompD* strain reaches a better systemic infection than the wild-type strain in BALB/c mice

**Figure 4:**
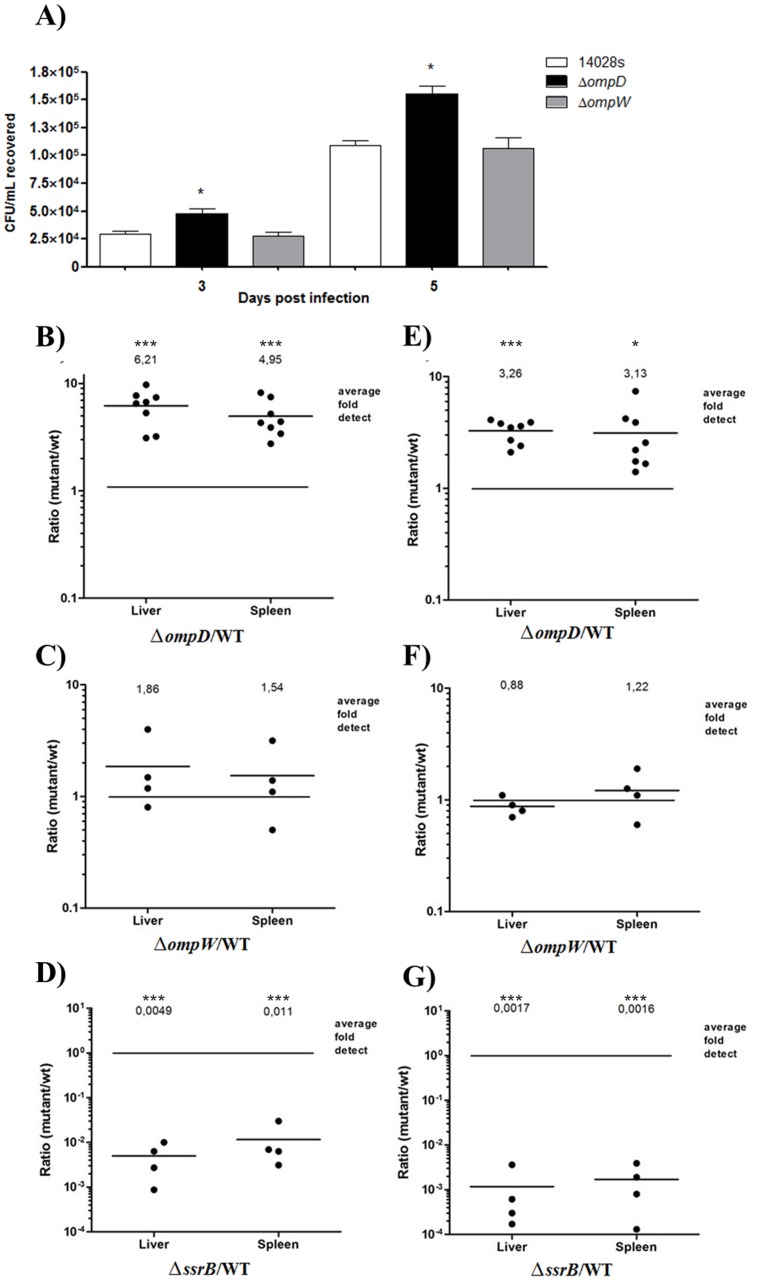
Effect of OmpD in systemic infection. (A) Bacteremia assays. Groups of six BALB/c mice were orally inoculated with 10^3^ bacteria of strains 14028 s, Δ*ompD* or Δ*ompW* (n = 6). CFU/ml were determined from samples of peripheral blood obtained after 3 and 5 days post infection. Values are mean ± SD. Asterisks represent statistical significant differences (T-test, * *p*≤0.05). (B to G) CI assays. A mixture (1∶1 ratio) of 10^3^ bacteria of strains 14028 s (WT) and Δ*ompD* (B and E), Δ*ompW* (C and F) or Δ*ssrB* (D and G) was orally (B, C and D) or intraperitoneally (E, F and G) inoculated to groups of six BALBc mice (n = 6). Liver and spleen were extracted from mice 5 days post infection and the CFU per g of organ was determined for the wild-type strain and the respective mutant. Each data point represents the competitive index (CI), which is the ratio of mutant/WT of recovered CFU/g tissue in the respective organ from each mouse. Horizontal bars represent the median values of ratios for the organ type.

To determine whether the detrimental effect of OmpD in the intracellular survival of *S*. Typhimurium is retained during the systemic infection, the bacteremia in mice at 3 and 5 days post-infection was assessed. For this purpose, groups of BALB/c mice were independently infected by the oral route with strains Δ*ompD*, Δ*ompW* or 14028 s at a dose of 10^3^ CFU and then bacteria were recovered from the circulating blood at various times to quantify the CFU (Figure 4A). The concentration of the Δ*ompD* strain in blood exceeded that of the wild-type strain by more than 2×10^4^ CFU/ml (2-fold increase). No difference in bacteremia was observed between the Δ*ompW* and the wild-type strains.

**Figure 5:**
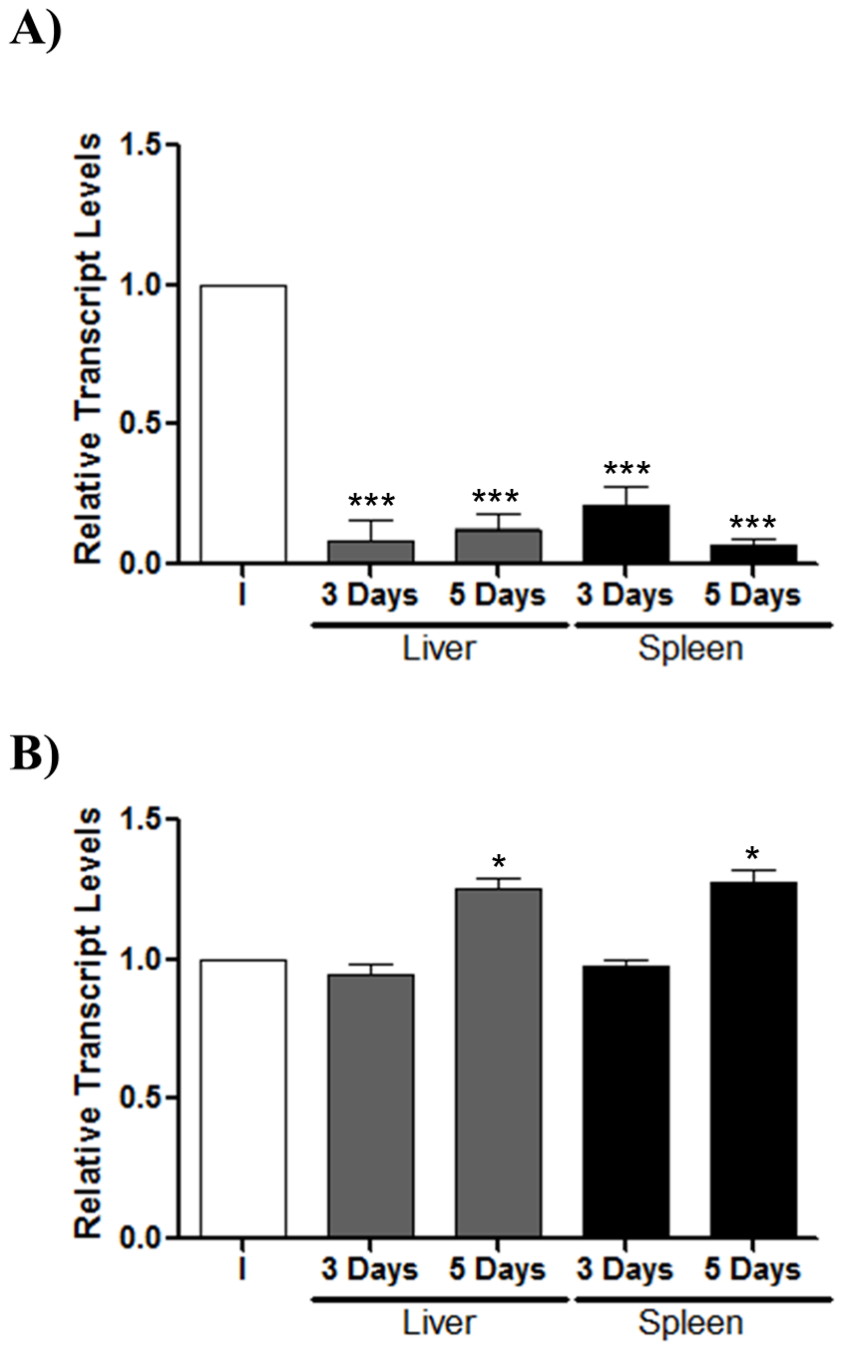
*ompD* expression in target organs from BALB/c mice infected with *S.* Typhimurium 14028 s. Bacteria were recovered from organs (liver and spleen) after three and five days post infection. The *ompD* (A) and *ompW* (B) transcript levels were measured by qRT-PCR and are indicated as relative transcript levels with respect to the levels of each gene in the bacterial inoculum (I) used for infecting mice (n = 3). Experiments were performed in biological and technical triplicate. Asterisks represent statistical differences between control and treated cells (* *p*≤0.05; *** *p*≤0.001). Values are mean ± SD.

As an accurate measure of virulence, the colonization of target organs was analyzed by means of CI assays. Mixed infection assays were performed using mice co-infected with strains Δ*ompD*, Δ*ompW* or Δ*ssrB* and 14028 s (1∶1 ratio), at total doses of 10^3^ bacteria. The CI for the Δ*ompD*/WT competitive mix in oral infection was 6.21 and 4.95-fold for the liver and spleen, respectively (Figure 4B). In intraperitoneal infections, the CI for these strains were 3.26 and 3.13-fold in the liver and spleen, respectively (Figure 4E). Thus, in both cases, the Δ*ompD* strain resulted more virulent than the wild-type strain. In contrast, for the Δ*ompW*/WT competitive mix, the CI values did not reflect differences in virulence, neither in the oral nor in the intraperitoneal infection (Figure 4C & F). As expected, the control mix Δ*ssrB*/WT evidenced highly attenuated virulence for the Δ*ssrB* strain (Figure 4D & G), as previously reported [37]. Taken together, these results confirm the deleterious effect of the OmpD porin in the survival of *Salmonella* during systemic infection. Hence, the possibility of a negative regulation of *ompD* expression when *S*. Typhimurium is colonizing target organs was evaluated by qRT-PCR in liver and spleen of mice infected with the wild-type strain, at 3 and 5 days post-infection. The *ompD* transcript levels decreased up to 80-fold in bacteria recovered from both liver and spleen, compared with its levels from the bacterial inoculum used for infecting the mice (Figure 5A). In contrast, the *ompW* transcript levels were similar or only increased slightly in bacteria recovered from organs with respect to those from the inoculum (Figure 5B). These results demonstrate that *ompD* expression is negatively regulated when *Salmonella* is in target organs during systemic infection.

## Discussion

Results from different assays performed in this study support a role for OmpD, but not for OmpW, in the intracellular proliferation of *Salmonella* in macrophages and in the systemic infection.

The role of the OmpD porin in virulence has remained controversial. OmpD inactivation in the virulent *Salmonella* strains SL1344 and UK-1 had no impact in the adherence or invasion of human intestine epithelial cells [19]. However, studies using purified OmpD from *S*. Typhimurium strains 14028 and 1826 blocked the attachment of bacteria to murine peritoneal macrophages [13], suggesting that OmpD is involved in the recognition of *S*. Typhimurium by host cells. Supporting this role, OmpD was shown to be involved in the adherence of *S*. Typhimurium to U937 monocytes and T84 intestine epithelial cells [20]. Our results showed that OmpD is not implicated in the adherence of *S*. Typhimurium to RAW264.7 macrophages (results are shown in Figure S1), which is consistent with previous reports using human intestine epithelial cells Int-407 macrophages [19]. Interestingly, in the gentamicin protection assay there was an increase in the invasion and proliferation of the Δ*ompD* strain in host cells as compared to the parental 14028 s wild-type strain (Figure 1A & B, respectively). This indicates that the OmpD porin has a negative effect in survival inside the macrophage. The observed differences in the role of OmpD in adherence, between previous literature and the present work, might be due to different factors including method of construction of *S*. Typhimurium *ompD* mutants (gene deletion vs transposon mutagenesis), cell lines used (RAW264.7 murine macrophages vs human epithelial cell lines [19]), and MOI used (10∶1 and 100∶1 vs 1000∶1 [20]).

Since OmpD inactivation increased survival inside macrophages, the *ompD* transcript levels were analyzed by qRT-PCR from wild-type bacteria residing in macrophages at different time points post infection (1 to 12 h), corresponding to the stages of adherence, invasion and proliferation. These assays showed a reduction of *ompD* transcript levels (Figure 2), suggesting that *Salmonella* decrease the expression of OmpD in order to increase its ability to survive intracellularly (Figure 2A). In this sense, it was previously shown that OmpD facilitates the uptake of H_2_O_2_ and the expression of *ompD* decreases in response to H_2_O_2_ treatment [7]. Hence, *ompD* down regulation could represent a bacterial strategy to evade, in part, the toxic effect of ROS or other bactericidal compounds generated by the macrophage, or both. Previous transcriptomic analyses of *S*. Typhimurium in macrophages (J774) also showed that the *ompD* (*nmpC*) transcript levels decreased 10-fold in comparison to the control [38].

Another observation that argues in favor of a detrimental effect of OmpD, is that the overexpression of the *ompD* gene produced a decrease in bacterial survival at 6, 8 and 12 h post-infection in macrophages (Figure 1 C–E). Hence, the increase in the proportion of this porin might elevate the uptake of toxic compounds such as ROS, produced within the SCV. Moreover, OmpD is recognized as a surface antigen during the immune response [21], so its increased production would result in an enhanced response against *Salmonella*. In fact, total ROS levels generated by macrophages infected with the Δ*ompD* strain were lower than those of macrophages infected with the wild-type strain at 5 h post infection in the proliferation stage (Figure 3A). The decrease in total ROS levels is consistent with higher intracellular survival (Figure 1) and with the fact that *ompD* expression is strongly down-regulated. The macrophage has a signaling lymphocyte-activation molecule (SLAM) which regulates the activity of the NADPH oxidase 2 complex (NOX2) and phagolysosomal maturation after entering the phagosome. SLAM interacts at least with the *E. coli* OmpC and OmpF porins [14], but given that OmpD is the most abundant outer membrane protein in *S*. Typhimurium [17], it is tempting to speculate that OmpD is implicated in a similar recognition mechanism within the SCV, and therefore trigger the ROS response. Supporting this hypothesis, our *in silico* analyses revealed that OmpD shares a similar electrostatic potential at the surface of the pore and a high sequence identity with OmpC and OmpF (Figure 3B and C). These properties could allow OmpD to interact with the surface of SLAM, which has a positive electrostatic potential. Together, this could explain, at least in part, why macrophages infected with a Δ*ompD* strain have diminished ROS levels (Figure 3A).

Dorman *et al*. [18] evaluated the virulence of a transposon insertion *ompD* mutant and found that it was less virulent than the respective wild-type strain, with an increase of 23-fold in oral LD_50_. Conversely, Meyer *et al*. [19] and later Selke *et al*. [44] did not find significant differences in the LD_50_ between a transposon insertion and an *ompD* mutant, respectively, and their parental wild-type strain. In this work, we determined the ability of a Δ*ompD* mutant to compete with its parental wild-type strain in colonizing liver and spleen of mice by competitive indices (CI) through oral and intraperitoneal inoculation routes. An increase in the CI of the Δ*ompD*/14028 s strains ratio at a low infective dose (10^3^ CFU, Figure 4), but not at higher doses (data not shown), was observed. This reflects a higher rate of dissemination of the *ompD* mutant in the mouse, as compared with the wild-type strain. Consistently, LD_50_ assays indicated that the *ompD* mutant showed a higher early lethality than the wild-type strain at lower doses of infection (10^3^–10^1^), but not at higher doses (data not shown). The dose-dependent effect observed for the Δ*ompD* strain in these assays could help to explain the discrepancies between the results previously reported about the role of OmpD in *Salmonella* virulence [18], [19], [44].

The OmpD protein and the *ompD* gene have been reported to be down-regulated in *S*. Typhimurium biofilms, leading to a decrease in membrane permeability and enhanced resistance to antimicrobial stresses [45], [46]. These observations support the concept that negative modulation of *ompD* expression at different stages of the infection cycle is crucial for the survival of *S*. Typhimurium in the host, as a mechanism of adaptation to adverse conditions. Furthermore, alterations in the ratio of surface membrane proteins could affect the expression of other proteins involved in virulence, such as proteins OmpS1, OmpS2, and PagK among others [15], [47].

## Supporting Information

Figure S1 **Effect of OmpD on the adherence of** ***S*** **. Typhimurium to macrophages.** Murine RAW 264.7 macrophages were infected (MOI of 100∶1) with *S*. Typhimurium 14028 s and its Δ*ompD*, Δ*ompW* or Δ*ssrB* derivative mutants. CFU of the different strains were determined after recovery from infected macrophages at the indicated time points. The relative percentage of adherence at 0,5 and 1 h post infection was determined to analyze the effect of the absence of *ompD*, *ompW* or *ssrB* genes.
(DOC)

Figure S2 **Sequence similarity network of OmpD and its closest homologues in the Omp superfamily.** Nodes represent protein sequences, and edges represent worst reciprocal blastp E-values that are higher than a given threshold. Visualization and color scheme as depicted in Figure 3.B. Edges filtered to e-value <1e-155, median alignment length: 380 residues, median identity: 65.2%.
(DOC)

## References

[1] Nikaido H (1996) Multidrug efflux pumps of gram-negative bacteria. J Bacteriol 178: 5853–5859. 8830678 10.1128/jb.178.20.5853-5859.1996 PMC178438

[2] Koebnik R, Locher KP, Van Gelder P (2000) Structure and function of bacterial outer membrane proteins: barrels in a nutshell. Mol Microbiol 37: 239–253. 10931321 10.1046/j.1365-2958.2000.01983.x

[3] Gil F, Ipinza F, Fuentes J, Fumeron R, Villarreal JM, et al (2007) The *ompW* (porin) gene mediates methyl viologen (paraquat) efflux in *Salmonella enterica* serovar typhimurium. Res Microbiol 158: 529–536. 17618087 10.1016/j.resmic.2007.05.004

[4] Santiviago CA, Fuentes JA, Bueno SM, Trombert AN, Hildago AA, et al (2002) The *Salmonella enterica* sv. Typhimurium *smvA*, *yddG* and *ompD* (porin) genes are required for the efficient efflux of methyl viologen. Mol Microbiol 46: 687–698. 12410826 10.1046/j.1365-2958.2002.03204.x

[5] Nikaido H (2003) Molecular basis of bacterial outer membrane permeability revisited Microbiol Mol Biol Rev. 67: 593–656. 10.1128/MMBR.67.4.593-656.2003 PMC309051 14665678

[6] Morales EH, Calderón IL, Collao B, Gil F, Porwollik S, et al (2012) Hypochlorous acid and hydrogen peroxide-induced negative regulation of *Salmonella enterica* serovar Typhimurium *ompW* by the response regulator ArcA. BMC Microbiol 12: 63. 22545862 10.1186/1471-2180-12-63 PMC3358236

[7] Calderón IL, Morales E, Caro NJ, Chahúan CA, Collao B, et al (2011) Response regulator ArcA of *Salmonella enterica* serovar Typhimurium downregulates expression of OmpD, a porin facilitating uptake of hydrogen peroxide. Res Microbiol 162: 214–222. 21144897 10.1016/j.resmic.2010.11.001

[8] Achouak W, Heulin T, Pagès JM (2001) Multiple facets of bacterial porins. FEMS Microbiol Lett 199: 1–7. 11356559 10.1111/j.1574-6968.2001.tb10642.x

[9] Holm MM, Vanlerberg SL, Foley IM, Sledjeski DD, Lafontaine ER (2004) The *Moraxella catarrhalis* porin-like outer membrane protein CD is an adhesin for human lung cells. Infect Immun 72: 1906–1913. 15039309 10.1128/IAI.72.4.1906-1913.2004 PMC375153

[10] Albertí S, Marqués G, Hernández-Allés S, Rubires X, Tomás JM, et al (1996) Interaction between complement subcomponent C1q and the *Klebsiella pneumoniae* porin OmpK36. Infect Immun 64: 4719–4725. 8890231 10.1128/iai.64.11.4719-4725.1996 PMC174437

[11] Bernardini ML, Sanna MG, Fontaine A, Sansonetti PJ (1993) OmpC is involved in invasion of epithelial cells by *Shigella flexneri* . Infect Immun 61: 3625–3635. 8359885 10.1128/iai.61.9.3625-3635.1993 PMC281057

[12] Liu YF, Yan JJ, Lei HY, Teng CH, Wang MC, et al (2012) Loss of outer membrane protein C in *Escherichia coli* contributes to both antibiotic resistance and escaping antibody-dependent bactericidal activity. Infect Immun 80: 1815–1822. 22354022 10.1128/IAI.06395-11 PMC3347438

[13] Negm RS, Pistole TG (1998) Macrophages recognize and adhere to an OmpD-like protein of *Salmonella* typhimurium. FEMS Immunol Med Microbiol 20: 191–199. 9566490 10.1111/j.1574-695X.1998.tb01127.x

[14] Berger SB, Romero X, Ma C, Wang G, Faubion WA, et al (2010) SLAM is a microbial sensor that regulates bacterial phagosome functions in macrophages. Nat Immunol 11: 920–927. 20818396 10.1038/ni.1931 PMC3338319

[15] Rodríguez-Morales O, Fernández-Mora M, Hernández-Lucas I, Vázquez A, Puente JL, et al (2006) *Salmonella enterica* serovar Typhimurium *ompS1* and *ompS2* mutants are attenuated for virulence in mice. Infect Immun 74: 1398–1402. 16428792 10.1128/IAI.74.2.1398-1402.2006 PMC1360296

[16] Santiviago CA, Toro CS, Bucarey SA, Mora GC (2001) A chromosomal region surrounding the *ompD* porin gene marks a genetic difference between *Salmonella* typhi and the majority of *Salmonella* serovars. Microbiology 147: 1897–1907. 11429466 10.1099/00221287-147-7-1897

[17] Santiviago CA, Toro CS, Hidalgo AA, Youderian P, Mora GC (2003) Global regulation of the *Salmonella* enterica serovar typhimurium major porin, OmpD. J Bacteriol 185: 5901–5905. 13129964 10.1128/JB.185.19.5901-5905.2003 PMC193956

[18] Dorman CJ, Chatfield S, Higgins CF, Hayward C, Dougan G (1989) Characterization of Porin and *ompR* Mutants of a Virulent Strain of *Salmonella* Typhimurium: *ompR* Mutants Are Attenuated in Vivo. Infec Immun 57: 2136–2140. 2543631 10.1128/iai.57.7.2136-2140.1989 PMC313852

[19] Meyer PN, Wilmes-Riesenberg MR, Stathopoulos C, Curtiss R (1998) Virulence of a *Salmonella* Typhimurium OmpD Mutant. Infect Immun 66: 387–390. 9423887 10.1128/iai.66.1.387-390.1998 PMC107915

[20] Hara-Kaonga B, Pistole TG (2004) OmpD but not OmpC is involved in adherence of *Salmonella enterica* serovar Typhimurium to human cells. Can J Microbiol 50: 719–727. 15644926 10.1139/w04-056

[21] Gil-Cruz C, Bobat S, Marshall JL, Kingsley RA, Ross EA, et al (2004) The porin OmpD from nontyphoidal *Salmonella* is a key target for a protective B1b cell antibody response. Proc Natl Acad Sci 106: 9803–9808. 10.1073/pnas.0812431106 PMC2701014 19487686

[22] Lissner CR, Swanson RN, O'Brien AD (1983) Genetic Control of the Innate Resistance of Mice to *Salmonella* Typhimurium: Expression of the Ity Gene in Peritoneal and Splenic Macrophages Isolated *in Vitro* . J Immunol 131: 3006–3013. 6358358

[23] Loessner H, Endmann A, Leschner S, Westphal K, Rohde M, et al (2007) Remote control of tumour-targeted *Salmonella enterica* serovar Typhimurium by the use of L-arabinose as inducer of bacterial gene expression *in vivo* . Cell Microbiol 9: 1529–1537. 17298393 10.1111/j.1462-5822.2007.00890.x

[24] Echave P, Tamarit J, Cabiscol E, Ros J (2003) Novel Antioxidant Role of Alcohol Dehydrogenase E from *Escherichia coli* . J Biol Chem 278: 30193–30198. 12783863 10.1074/jbc.M304351200

[25] Segura I, Casadesús J, Ramos-Morales F (2004) Use of mixed infections to study cell invasion and intracellular proliferation of *Salmonella enterica* in eukaryotic cell cultures. J Microbiol Methods 56: 83–91. 14706753 10.1016/j.mimet.2003.09.004

[26] Torii I, Oka S, Hotomi M, Benjamin WH, Takai T, et al (2008) PIR-B-deficient mice are susceptible to *Salmonella* infection. J Immunol 181: 4229–4239. 18768880 10.4049/jimmunol.181.6.4229 PMC2613810

[27] Pfaffl MW (2001) A New Mathematical Model for Relative Quantification in Real-time RT-PCR. Nucleic Acids Res 29: e45. 11328886 10.1093/nar/29.9.e45 PMC55695

[28] UniProt Consortium (2014) Activities at the Universal Protein Resource (UniProt). Nucleic Acids Res 42: D191–D198. 24253303 10.1093/nar/gkt1140 PMC3965022

[29] Li W, Godzik A (2006) Cd-hit: a fast program for clustering and comparing large sets of protein or nucleotide sequences. Bioinformatics 22: 1658–1659. 16731699 10.1093/bioinformatics/btl158

[30] Altschul SF, Gish W, Miller W, Myers EW, Lipman DJ (1990) Basic local alignment search tool. J Mol Biol 215: 403–410. 2231712 10.1016/S0022-2836(05)80360-2

[31] Cline MS, Smoot M, Cerami E, Kuchinsky A, Landys N, et al (2007) Integration of biological networks and gene expression data using Cytoscape. Nat Protoc 2: 2366–2382. 17947979 10.1038/nprot.2007.324 PMC3685583

[32] Sali A, Blundell TL (1993) Comparative protein modelling by satisfaction of spatial restraints. Journal of Molecular Biology 234: 779–815. 8254673 10.1006/jmbi.1993.1626

[33] Eargle J, Wright D, Luthey-Schulten Z (2006) Multiple Alignment of protein structures and sequences for VMD. Bioinformatics 22: 504–506. 16339280 10.1093/bioinformatics/bti825

[34] Baker N, Sept D, Joseph S, Holst M, McCammon JA (2001) Electrostatics of nanosystems: application to microtubules and the ribosome. Proc. Natl. Acad. Sci. U.S.A 98: 10037–10041. 11517324 10.1073/pnas.181342398 PMC56910

[35] Humphrey W, Dalke A, Schulten K (1996) VMD: visual molecular dynamics. J Mol Graph 14: : 33–8, 27–8. 10.1016/0263-7855(96)00018-5 8744570

[36] Sanner M, Olson A, Spehner J (1996) Reduced surface: an efficientway to compute molecular surfaces. Biopolymers 38(3): 305–20. 8906967 10.1002/(SICI)1097-0282(199603)38:3%3C305::AID-BIP4%3E3.0.CO;2-Y

[37] Feng X, Walthers D, Oropeza R, Kenney LJ (2004) The Response Regulator SsrB Activates Transcription and Binds to a Region Overlapping OmpR Binding Sites at Salmonella Pathogenicity Island 2. Mol Microbiol 54: 823–835. 15491370 10.1111/j.1365-2958.2004.04317.x

[38] Eriksson S, Lucchini S, Thompson A, Rhen M, Hinton JC (2003) Unravelling the Biology of Macrophage Infection by Gene Expression Profiling of Intracellular Salmonella Enterica. Mol Microbiol 47: 103–118. 12492857 10.1046/j.1365-2958.2003.03313.x

[39] Mastroeni P, Vazquez-Torres A, Fang FC, Xu Y, Khan S, et al (2000) Antimicrobial actions of the NADPH phagocyte oxidase and inducible nitric oxide synthase in experimental salmonellosis. II. Effects on microbial proliferation and host survival in vivo. J Exp Med 192: 237–248. 10899910 10.1084/jem.192.2.237 PMC2193252

[40] Vazquez-Torres A, Jones-Carson J, Mastroeni P, Ischiropoulos H, Fang FC (2000) Antimicrobial actions of the NADPH phagocyte oxidase and inducible nitric oxide synthase in experimental salmonellosis. I. Effects on microbial killing by activated peritoneal macrophages in vitro. J Exp Med 192: 227–236. 10899909 10.1084/jem.192.2.227 PMC2193262

[41] Atkinson HJ, Morris JH, Ferrin TE, Babbitt PC (2009) Using sequence similarity networks for visualization of relationships across diverse protein superfamilies. PLoS One 4: e4345. 19190775 10.1371/journal.pone.0004345 PMC2631154

[42] Lukk T, Sakai A, Kalyanaraman C, Brown SD, Imker HJ, et al (2012) Homology models guide discovery of diverse enzyme specificities among dipeptide epimerases in the enolase superfamily. Proc Natl Acad Sci U S A 109: 4122–4127. 22392983 10.1073/pnas.1112081109 PMC3306705

[43] Brown SD, Babbitt PC (2012) Inference of functional properties from large-scale analysis of enzyme superfamilies. J Biol Chem 287: 35–42. 22069325 10.1074/jbc.R111.283408 PMC3249087

[44] Selke M, Meens J, Springer S, Frank R, Gerlach GF (2007) Immunization of Pigs to Prevent Disease in Humans: Construction and Protective Efficacy of a *Salmonella Enterica* Serovar Typhimurium Live Negative-marker Vaccine. Infect Immun 75: 2476–2483. 17296750 10.1128/IAI.01908-06 PMC1865763

[45] He X, Ahn J (2011) Survival and virulence properties of multiple antibiotic-resistant *Salmonella* Typhimurium under simulated gastrointestinal conditions. Int J Food Sci Tech 46: 2164–2172.

[46] Zou Y, Woo J, Ahn J (2012) Cellular and molecular responses of *Salmonella* Typhimurium to antimicrobial-induced stresses during the planktonic-to-biofilm transition. Lett Appl Microbiol 55: 274–282. 22803575 10.1111/j.1472-765X.2012.03288.x

[47] Yoon H, Ansong C, Adkins JN, Heffron F (2011) Discovery of *Salmonella* virulence factors translocated via outer membrane vesicles to murine macrophages. Infect Immun 79: 2182–2192. 21464085 10.1128/IAI.01277-10 PMC3125828

