# Supplementary material for: Participation of the Salmonella OmpD Porin in the Infection of RAW264.7 Macrophages and BALB/c Mice
Source: PLoS One. 2014 Oct 31;9(10):e111062. doi: 10.1371/journal.pone.0111062 (PMC4215857; doi:10.1371/journal.pone.0111062)
Supplement: Figure S1 — Effect of OmpD on the adherence of S . Typhimurium to macrophages. Murine RAW 264.7 macrophages were infected (MOI of 100∶1) with S. Typhimurium 14028 s and its ΔompD, ΔompW or ΔssrB derivative mutants. CFU of the different strains were determined after recovery from infected macrophages at the indicated time points. The relative percentage of adherence at 0,5 and 1 h post infection was determined to analyze the effect of the absence of ompD, ompW or ssrB genes. (DOC) [file pone.0111062.s001.doc]

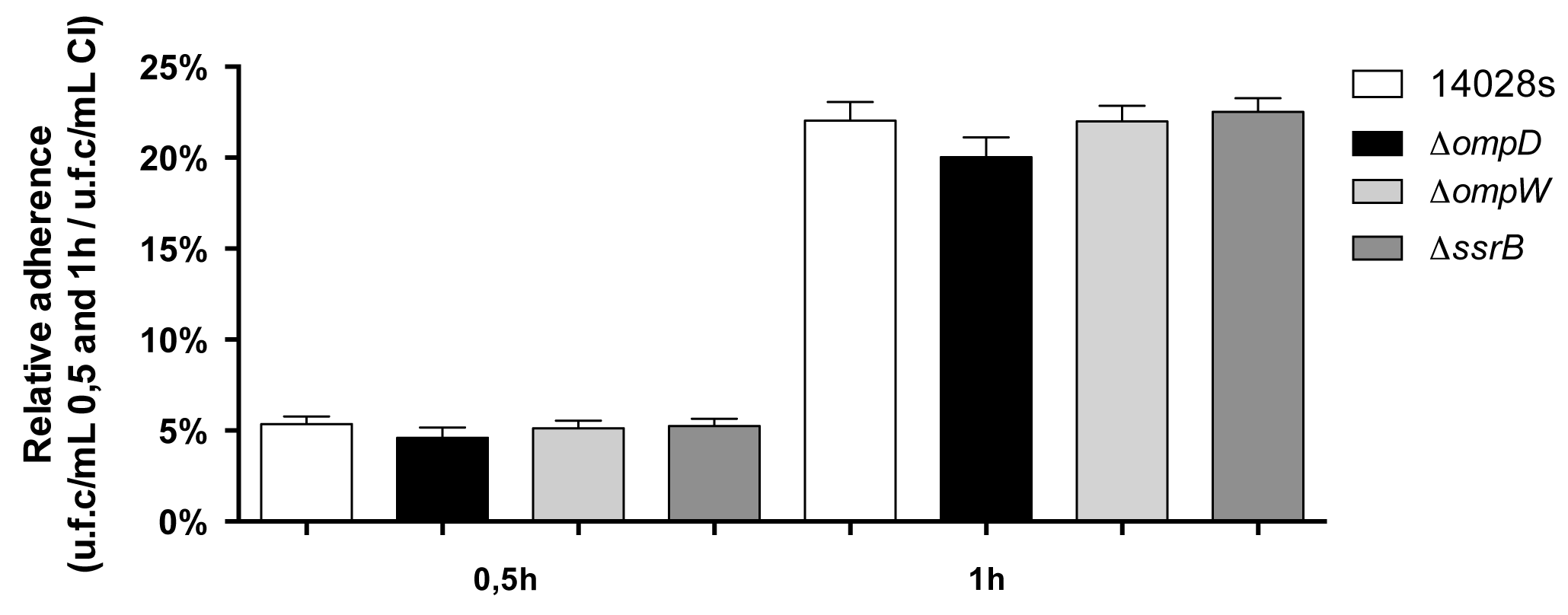


**Figure S1. Effect of OmpD on the adherence of *S*. Typhimurium to macrophages.** Murine RAW 264.7 macrophages were infected (MOI of 100:1) with *S*. Typhimurium 14028s and its ∆*ompD,* ∆*ompW* or ∆*ssrB* derivative mutants. CFU of the different strains were determined after recovery from infected macrophages at the indicated time points. The relative percentage of adherence at 0,5 and 1 h post infection was determined to analyze the effect of the absence of *ompD*, *ompW* or *ssrB* genes.
