# Supplementary material for: Participation of the Salmonella OmpD Porin in the Infection of RAW264.7 Macrophages and BALB/c Mice
Source: PLoS One. 2014 Oct 31;9(10):e111062. doi: 10.1371/journal.pone.0111062 (PMC4215857; doi:10.1371/journal.pone.0111062)
Supplement: Figure S2 — Sequence similarity network of OmpD and its closest homologues in the Omp superfamily. Nodes represent protein sequences, and edges represent worst reciprocal blastp E-values that are higher than a given threshold. Visualization and color scheme as depicted in Figure 3.B. Edges filtered to e-value <1e-155, median alignment length: 380 residues, median identity: 65.2%. (DOC) [file pone.0111062.s002.doc]

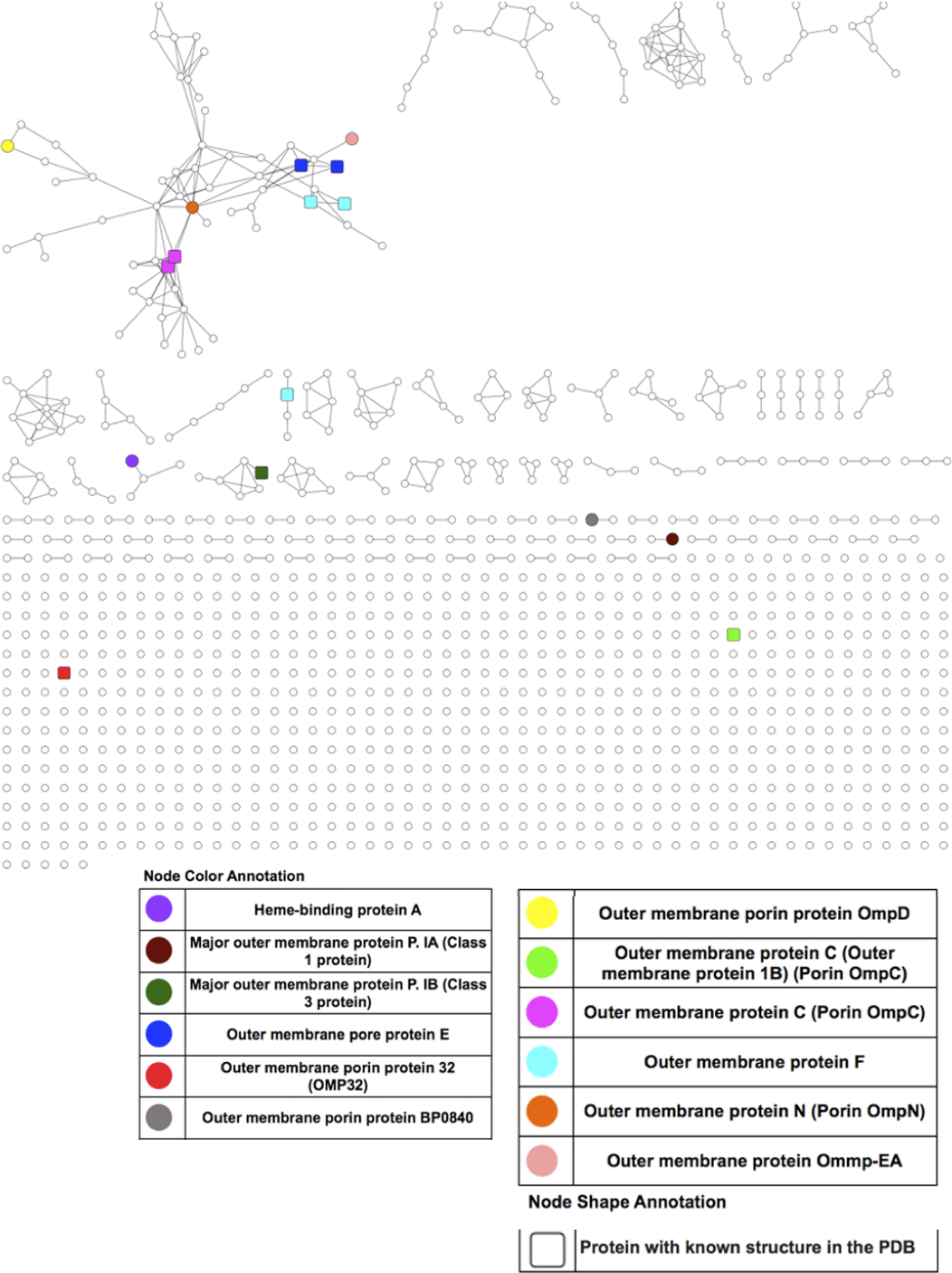


**Figure S2. Sequence similarity network of OmpD and its closest homologues in the Omp superfamily.** Nodes represent protein sequences, and edges represent worst reciprocal blastp E-values that are higher than a given threshold. Visualization and color scheme as depicted in Figure 3.B. Squares correspond to proteins that have known crystal structures in the Protein Data Bank. Edges filtered to e-value < 1e-155, median alignment length: 380 residues, median identity: 65.2%.
